# Pigmented polypoid basal cell carcinoma: a rare clinicopathological variant^⋆^

**DOI:** 10.1016/j.abd.2023.03.011

**Published:** 2024-06-08

**Authors:** Bruno de Carvalho Fantini, Cecilia Anatriello dos Santos, Sebastião Antônio de Barros Junior, Cacilda da Silva Souza

**Affiliations:** a Department of Internal Medicine, Ribeirão Preto Faculty of Medicine, Universidade de São Paulo, Ribeirão Preto, SP, Brazil; b Department of Pathology and Forensic Medicine, Ribeirão Preto Faculty of Medicine Universidade de São Paulo, Ribeirão Preto, SP, Brazil

*Dear Editor,*

Polypoid basal cell carcinoma (BCC) is a rare entity that is clinically distinct from other BCC subtypes, as it is pedunculated and connected by a stalk to the surface of the skin, and histopathology, exhibits tumor aggregates restricted to the exophytic polypoid area.^1^

A 69-year-old Caucasian man reported a rapidly growing pigmented lesion, about a year ago, on the lateral side of his right leg (Fig. 1), which he associated with local trauma. He denied excessive sun exposure. On dermatological examination, he had a tumor with an erythematous and shiny surface in the center, and pigmented on the periphery, measuring 40 mm in its largest diameter, pedunculated, transluminescent and of fibrous consistency (Fig. 2A). Dermoscopy showed large blue-gray ovoid nests on the periphery of the lesion and short white lines (chrysalises) across the entire surface, but without arboriform telangiectasias (Fig. 2B). There were no lymph node enlargements. Following excision, histopathology showed, in a panoramic view, a polypoid tumor consisting of basaloid neoplastic aggregations with peripheral palisading, varying in size, shape and pigment distribution, limites to the upper and middle part of the polyp (Fig. 3; Fig. 4A-B). The immunohistochemical markers Melan-A and HMB45 were negative. It was concluded that it was a nodular, cribriform, cystic, pigmented basal cell carcinoma with free surgical margins. The option for closure by secondary intention until diagnostic confirmation resulted in good evolution, with no signs of recurrence or metastasis up to three months of follow-up.

**Figure 1:**
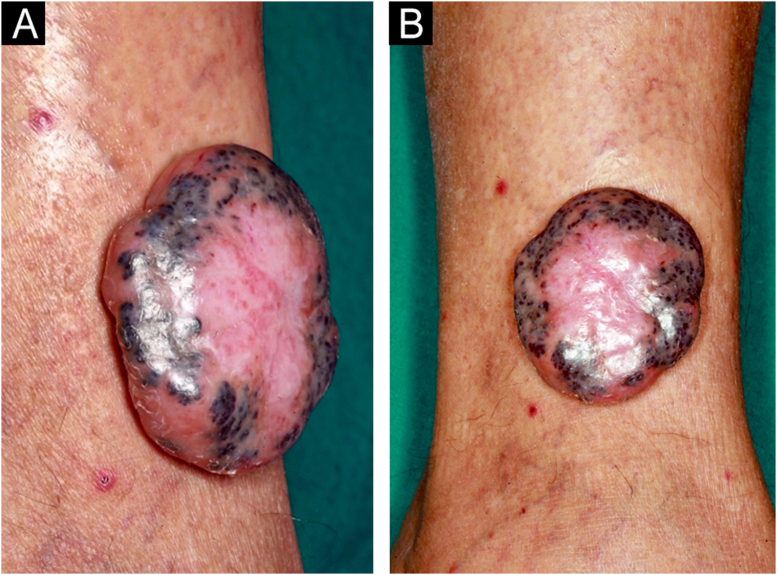
(A‒B) Lateral and frontal view of the exophytic pedunculated tumor, 40-mm in its largest diameter, showing an erythematous, shiny, pearly surface, and pigmented areas on the periphery.

**Figure 2:**
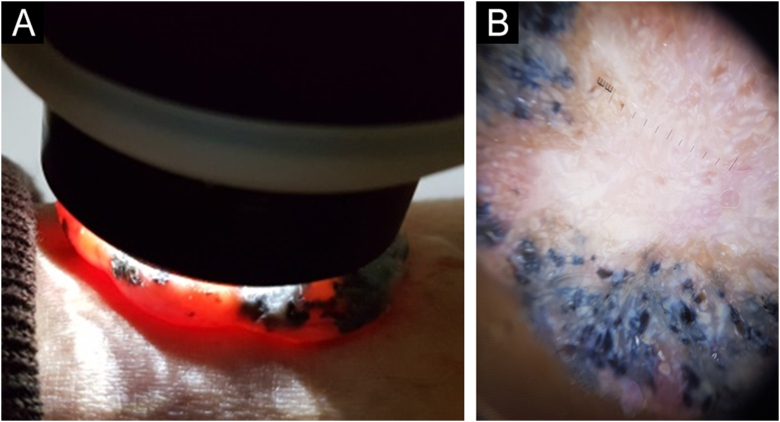
(A) Transillumination testing of the polypoid tumor. (B) On dermoscopy, large blue-grey ovoid nests on the periphery of the lesion can be observed, with white shiny areas and lines (chrysalises) predominating in the center, without arboriform telangiectasias.

**Figure 3:**
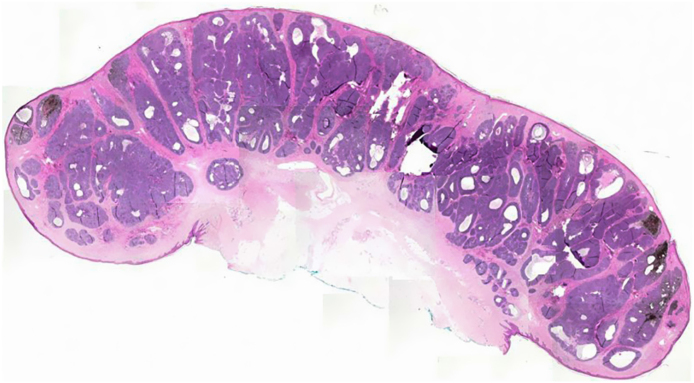
On histopathology, a panoramic view showed a polypoid tumor consisting of basaloid neoplastic aggregations of varyng sizes and shapes with peripheral palisading, limited to the upper and middle part of the polyp.

**Figure 4:**
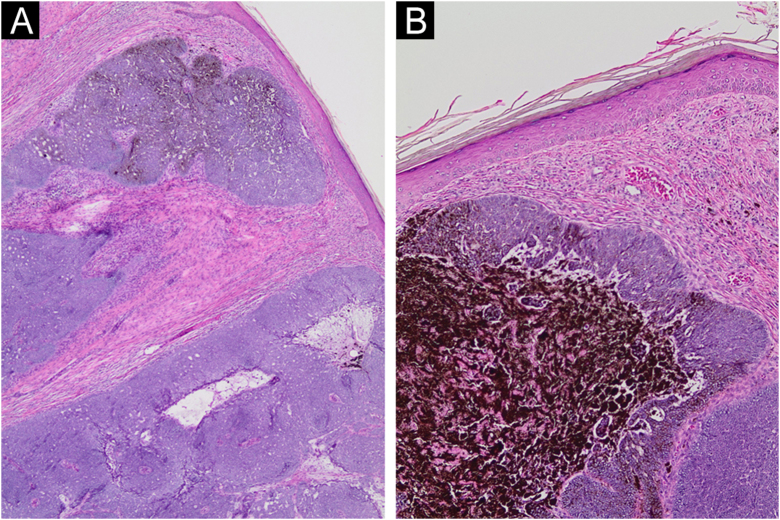
(A‒B) Detail of basaloid neoplastic aggregations with peripheral palisading, and areas of melanin deposition (Hematoxylin & eosin, ×40, ×100, respectively).

The combination of reviews in the English and Japanese literature recorded just over 30 cases. In these reviews, the tumors varied between 10 mm and 80 mm in their largest diameter, and the size of the reported polyp was considered large.^2^, ^3^

Despite their large size, most lesions showed well-circumscribed nodules, without an aggressive infiltration pattern, and the neoplasm were restricted to the polypoid area. Distinctly, these polypoid BCCs predominated on the scalp and in the genital, perianal, or gluteal regions; followed by the trunk, face and perioptic regions, with 13% of cases found in the extremities.^3^, ^4^

The polypoid, sessile, or pedunculated shape of the neoplasm must be differentiated from Pinkus fibroepithelioma, a variant of the spectrum between BCC and trichoblastoma, presenting a peculiar and unmistakable histopathology.^5^

In conclusion, polypoid BCC has been recognized as a variant of nodular BCC based on its clinical, morphological, and histopathological peculiarities; additionally, its preferential locations suggest other etiological factors, in addition to the recognized exposure to ultraviolet radiation associated with BCCs.^1^, ^2^, ^3^, ^4^

## Financial support

None declared.

## Authors' contributions

Bruno de Carvalho Fantini: Design of the case study, data survey, collection, or analysis and interpretation of data; intellectual participation in the propaedeutic and/or therapeutic conduct of the studied case; approval of the final version of the manuscript.

Cecilia Anatriello dos Santos: Data survey, collection, or analysis, and interpretation of data; intellectual participation in the propaedeutic and/or therapeutic conduct of the studied case; approval of the final version of the manuscript.

Sebastião Antônio de Barros Junior: Data survey, collection, or analysis and interpretation of data; approval of the final version of the manuscript.

Cacilda da Silva Souza: Design and planning of the studied case; data survey, collection or analysis, and interpretation of data; drafting and editing of the manuscript or critical review of intellectual content; intellectual participation in the propaedeutic and/or therapeutic conduct of the studied case; critical review of the literature; approval of the final version of the manuscript.

## Conflicts of interest

None declared.
